# Identification of the shared gene signature and biological mechanism between type 2 diabetes and colorectal cancer

**DOI:** 10.3389/fgene.2023.1202849

**Published:** 2023-10-09

**Authors:** Xianqiang Liu, Dingchang Li, Wenxing Gao, Wen Zhao, Lujia Jin, Peng Chen, Hao Liu, Yingjie Zhao, Guanglong Dong

**Affiliations:** ^1^ Medical School of Chinese PLA, Beijing, China; ^2^ Department of General Surgery, The First Medical Center, Chinese PLA General Hospital, Beijing, China; ^3^ School of Medicine, Nankai University, Tianjin, China

**Keywords:** colorectal cancer, weighted gene co-expression network analysis, type 2 diabetes, bioinformatics analysis, hub gene signature

## Abstract

**Background:** The correlation of type 2 diabetes mellitus (T2DM) with colorectal cancer (CRC) has garnered considerable attention in the scientific community. Despite this, the molecular mechanisms underlying the interaction between these two diseases are yet to be elucidated. Hence, the present investigation aims to explore the shared gene signatures, immune profiles, and drug sensitivity patterns that exist between CRC and T2DM.

**Methods:** RNA sequences and characteristics of patients with CRC and T2DM were retrieved from The Cancer Genome Atlas and Gene Expression Omnibus databases. These were investigated using weighted gene co-expression network analysis (WGCNA) to determine the co-expression networks linked to the conditions. Genes shared between CRC and T2DM were analyzed by univariate regression, followed by risk prognosis assessment using the LASSO regression model. Various parameters were assessed through different software such as the ESTIMATE, CIBERSORT, AND SSGSEA utilized for tumor immune infiltration assessment in the high- and low-risk groups. Additionally, pRRophetic was utilized to assess the sensitivity to chemotherapeutic agents in both groups. This was followed by diagnostic modeling using logistic modeling and clinical prediction modeling using the nomogram.

**Results:** WGCNA recognized four and five modules that displayed a high correlation with T2DM and CRC, respectively. In total, 868 genes were shared between CRC and T2DM, with 14 key shared genes being identified in the follow-up analysis. The overall survival (OS) of patients in the low-risk group was better than that of patients in the high-risk group. In contrast, the high-risk group exhibited higher expression levels of immune checkpoints The Cox regression analyses established that the risk-score model possessed independent prognostic value in predicting OS. To facilitate the prediction of OS and cause-specific survival, the nomogram was established utilizing the Cox regression model.

**Conclusion:** The T2DM + CRC risk-score model enabled independent prediction of OS in individuals with CRC. Moreover, these findings revealed novel genes that hold promise as therapeutic targets or biomarkers in clinical settings.

## Introduction

The rising incidence of type 2 diabetes (T2DM) and colorectal cancer (CRC) has led to their recognition as major public health concerns, owing to the escalating mortality and morbidity rates associated with these diseases. T2DM is a metabolic disease characterized by insulin resistance and hyperinsulinemia that results in persistent chronic inflammation and metabolic disorders, such as pancreatic cancer (Wang et al., 2022), periodontitis (Chen et al., 2022). Reportedly, the condition affects over 450 million individuals globally, and this number is projected to increase to 642 million by 2040 (Ogurtsova et al., 2017). Besides inducing metabolic disorders and vascular damage, T2DM is also a major predisposing factor for cancer, primarily gastrointestinal malignancies, such as CRC (Calle et al., 2003). Globally, CRC is the third most prevalent malignant gastrointestinal cancer with the second highest cancer-linked mortality rate (Siegel et al., 2020). The American Cancer Society has estimated that almost 151,030 new cases of CRC are expected to occur in 2022, with an estimated 52,580 deaths in both sexes. Furthermore, the incidence of CRC is projected to rise further (Siegel et al., 2022). The 5-year survival rate of individuals with advanced CRC is merely 15% (Bray et al., 2018; Ferlay et al., 2019). Recently, epidemiological reports have revealed a link between T2DM and CRC cancer (Kramer et al., 2012). Specifically, individuals with diabetes exhibit a higher relative risk of CRC by approximately 30% compared to those without diabetes. Moreover, diabetes has been shown to increase the risk of CRC recurrence (Meyerhardt et al., 2003). A meta-analysis by Zhu *et al.* (Zhu et al., 2017) revealed that the overall survival (OS) was 5 years shorter in the CRC + T2DM cohort than in the CRC cohort. In individuals with T2DM, the poor prognosis of CRC may be associated with the dysregulation of the cell proliferation cycle (Eguchi et al., 2022). Therefore, assessing the underlying mechanisms of T2DM and CRC is crucial for developing novel therapeutic approaches.

Obesity and a sedentary lifestyle are among the shared characteristics of CRC and T2DM, indicating a close relationship between the two conditions (Jin, 2008; Zelenko and Gallagher, 2014). Recent studies suggest that T2DM is a systemic chronic inflammatory disease linked to genetic alterations leading to recurrent damage and repair, upregulated cell proliferation, and ultimately CRC. In individuals with CRC, T2DM-induced cellular over-proliferation along with increased oxidative stress results in DNA damage and affects the repair of oncogenic DNA. This T2DM-related mechanism may be a major factor affecting the survival and prognosis of individuals with CRC (Stanich et al., 2011; Cho et al., 2016). Since T2DM and CRC have similar causal factors, such as high-calorie intake, high-fat diet, overweight, and sedentary lifestyle, it is common for diabetes and CRC to coexist clinically. Moreover, these two conditions can affect and promote each other, leading to disease progression (Potter, 1999; Expert Panel on Detection et al., 2001).

Currently, there is a lack of highly specific and sensitive biomarkers for CRC diagnosis and prognosis prediction in individuals with T2DM. Moreover, exploring the molecular pathways and networks linked to T2DM and CRC is essential for the development of patient screening, prevention, diagnosis, and treatment strategies. Therefore, in this research, a bioinformatics approach was employed to identify the interactions between T2DM and CRC.

## Materials and methods

### Dataset information

According to the pre-defined criteria, the GEO datasets (GSE17536, GSE17537, GSE7014, and GSE39582) and The Cancer Genome Atlas (TCGA)-CRC were chosen for this research. The summarized data of all four datasets are displayed in Table 1. Furthermore, GSE7014 and GSE39582 were paired into a discovery cohort for the weighted gene co-expression network analysis (WGCNA). GSE17536, GSE17537, and TCGA-CRC were selected into the validated cohorts for the analysis.

**TABLE 1:** Summary of three GEO datasets and TCGA dataset on patients with T2DM and CRC.

ID	Dataset	Platform	Samples	Disease	Group
1	GSE7014	GPL570	20 patients and 6 controls	T2DM	Discovery cohort
2	GSE39582	GPL570	566 patients and 19 controls	CRC	Discovery cohort
3	GSE17537	GPL570	177 patients	CRC	Validation cohort
4	GSE17536	GPL570	55 patients	CRC	Validation cohort
5	TCGA-CRC	Illumina	556 Patients and 28 controls	CRC	Validation cohort

CRC, colorectal cancer; GEO, gene expression omnibus; T2DM, Type 2 diabetes mellitus; TCGA, the cancer genome atlas.

### Immune infiltration analysis

The R package ESTIMATE (Version 1.0.13) was used to examine the tumor microenvironment (TME) of every individual afflicted with CRC by determining the stromal and immune cell fractions based on the gene expression signatures in tumor samples. The immune score (degree of immune cell infiltration), stromal score (stromal content), tumor purity, and ESTIMATE score (synthetic mark of stroma and immune) were assessed using ESTIMATE (Yoshihara et al., 2013). CIBERSORT was used to compute cell composition as per gene expression profiles. This process was employed for the calculation of the proportions of 22 immune cell types in each CRC individual (Newman et al., 2015). The sum of all fractions of these immune cells in each sample was 1. Additionally, the infiltration degree of 28 immune cell types was determined using the R package GSVA (Version 1.42.0) single-sample gene set enrichment analysis (SSGSEA) (Hanzelmann et al., 2013), based on the expression of genes in the published 28 gene sets for immune cells (Bindea et al., 2013).

### Development and assessment of nomograms and calibration curves

A nomogram model was developed using the 14 diabetes-associated mRNAs to predict 1-, 3-, and 5-year OS through the rms (Version 6.4.1) R package. Afterward, the calibration curves of the aforementioned OS were used to validate the performance of nomogram models using the bootstrap method with 1,000 resamples.

### WGCNA

WGCNA is an algorithm for analyzing gene expression patterns in numerous samples. This algorithm can cluster genes and construct modules based on similar gene expression patterns. Additionally, it can identify associations between these modules and biological characteristics (Langfelder and Horvath, 2008). This research utilized the R package WGCNA (Version 1.72.1) to develop networks of genes co-expressed between T2DM and CRC. Initially, a soft-threshold beta value of 12 for T2DM and 8 for CRC along with the gene-gene correlation matrix was used to build the adjacency matrix, which describes the strength of association between the nodes. Following the conversion of the constructed matrix into the topological overlap matrix, a gene hierarchical clustering dendrogram was established. This dendrogram enabled the identification of co-expression modules. Ultimately, the module eigengenes (MEs) were calculated, and their correlation with clinical characteristics was assessed to determine the disease-related modules.

### Cox regression analysis and least absolute shrinkage and selection operator (LASSO) for prognostic gene selection

To assess survival-linked genes, univariate Cox regression analysis was conducted for differentially expressed genes (DEGs). Simultaneously, the gene signature was established through the LASSO Cox regression model (R package “glmnet” (Version 4.1.7)) in order to avoid overfitting and enhance the credibility of screening out core genes (Friedman et al., 2010). Moreover, the selection of relevant features and quantification of the hazard ratios (HRs) with 95% confidence intervals (CIs) were conducted through multivariable Cox regression analyses (Vrieze, 2012). Genes with a *p*-value of <0.05 were regarded as suitable for further analysis.

### Drug sensitivity prediction

This research utilized the biggest pharmacogenomics database, the Genomics of Drug Sensitivity in Cancer (GDSC) (https://www.cancerrxgene.org), which is publicly available. Herein, the chemotherapeutic sensitivity for each tumor sample was predicted using the R package “pRRophetic (Version 0.5).” Ridge regression was utilized to estimate the half-maximal inhibitory concentration (IC50) of each tumor sample treated with a certain chemotherapy drug. With the GDSC training set, 10-fold cross-validation was employed to assess the accuracy of the prediction (Geeleher et al., 2014).

## Results

### Weighted gene co-expression modules in T2DM and CRC

**FIGURE 1:**
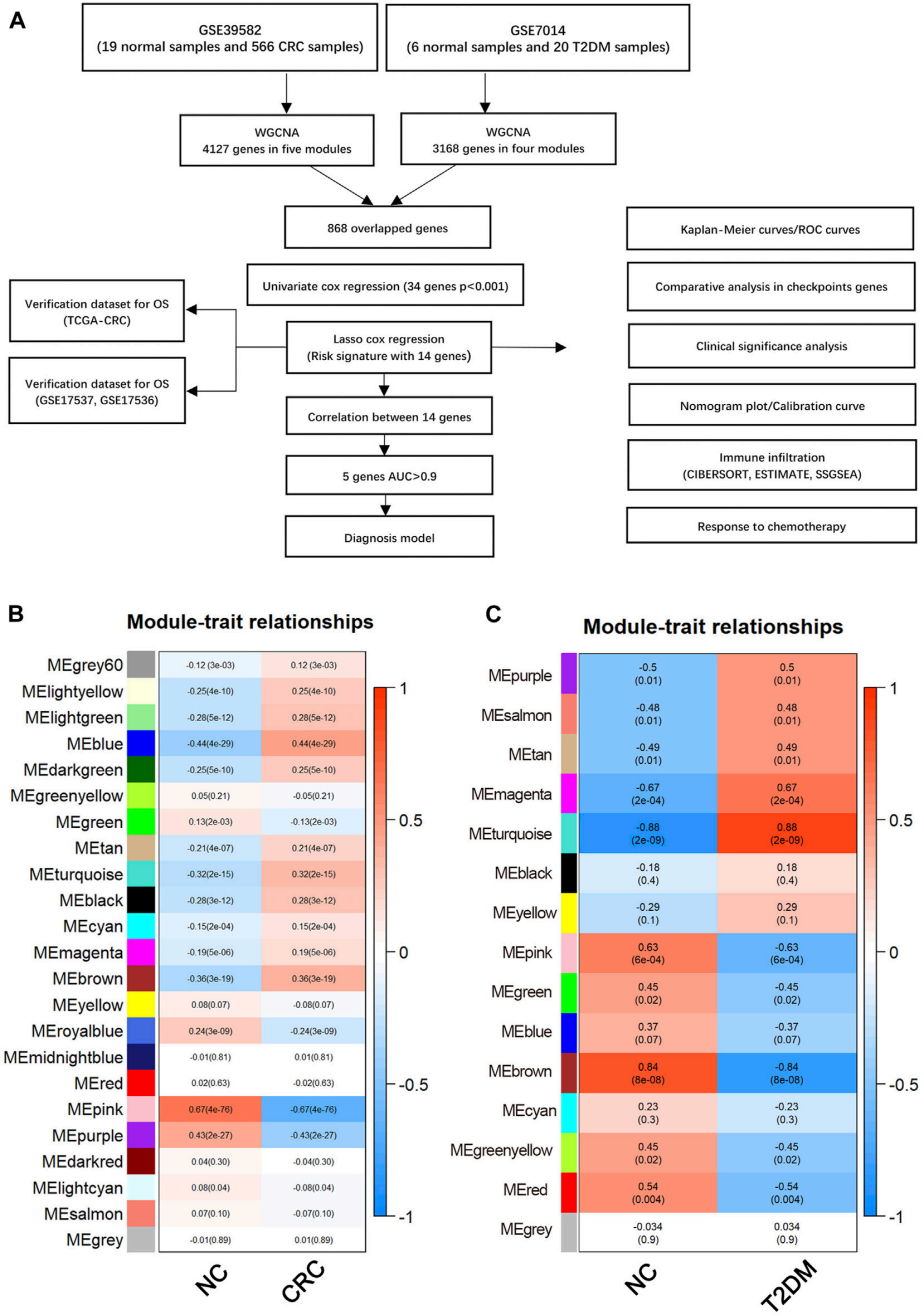
**(A)** Flow chart of this study. Weighted gene co-expression network analysis (WGCNA). **(B)** Module-trait relationships in CRC. Each image element contains the corresponding correlation and *p*-value. **(C)** Module-trait relationships in T2DM. Each pixel contains the corresponding correlation and *p*-value. CRC, Colorectal cancer; T2DM, Type 2 diabetes mellitus.

The development process of the risk model and further analyses are explained in detail (Figure 1A). Initially, using the WGCNA, 15 modules were identified in GSE7014, where each module was represented by a different color Next, a heatmap was established to examine the correlation between each module and the disease, by mapping module-trait relationships based on the Spearman correlation coefficient. (Figure 1B). Among the modules, four modules, including the “magenta,” “turquoise,” “pink,” and “brown,” exhibited a strong link to T2D and were identified as T2D-related modules (magenta module: r = −0.67, *p* = 0.00021; turquoise module: r = −0.88, *p* = 2.10e−09; pink module: r = 0.63, *p* = 0.0006, brown module: r = 0.84, *p* = 8.22e−08). The pink and brown modules, consisting of 328 and 1,022 genes respectively, showed a positive correlation with T2D. On the other hand, the magenta and turquoise modules, consisting of 184 and 1,634 genes respectively, exhibited a negative correlation with T2D.

After performing WGCNA on GSE39582, 23 modules were detected. Five modules, namely “blue”, “turquoise”, “purple”, “pink” and “brown,” with a high correlation with CRC were identified and selected as CRC-related modules (blue module: r = −0.44, *p* = 4.20e−09; turquoise module: r = −0.32, *p* = 1.67e−09; pink module: r = 0.66, *p* = 4.30e−76, brown module: r = −0.36, *p* = 3.01e−19; purple module: r = 0.43, *p* = 1.60e−27). The pink and purple modules, comprised of 446 and 234 genes, respectively, showed a positive correlation with CRC. On the other hand, comprised of 1,092, 1,071, and 1,284 genes, respectively, the blue, brown, and turquoise modules exhibited a negative correlation with CRC (Figure 1C).

### Construction and validation of the shared gene risk signature

**FIGURE 2:**
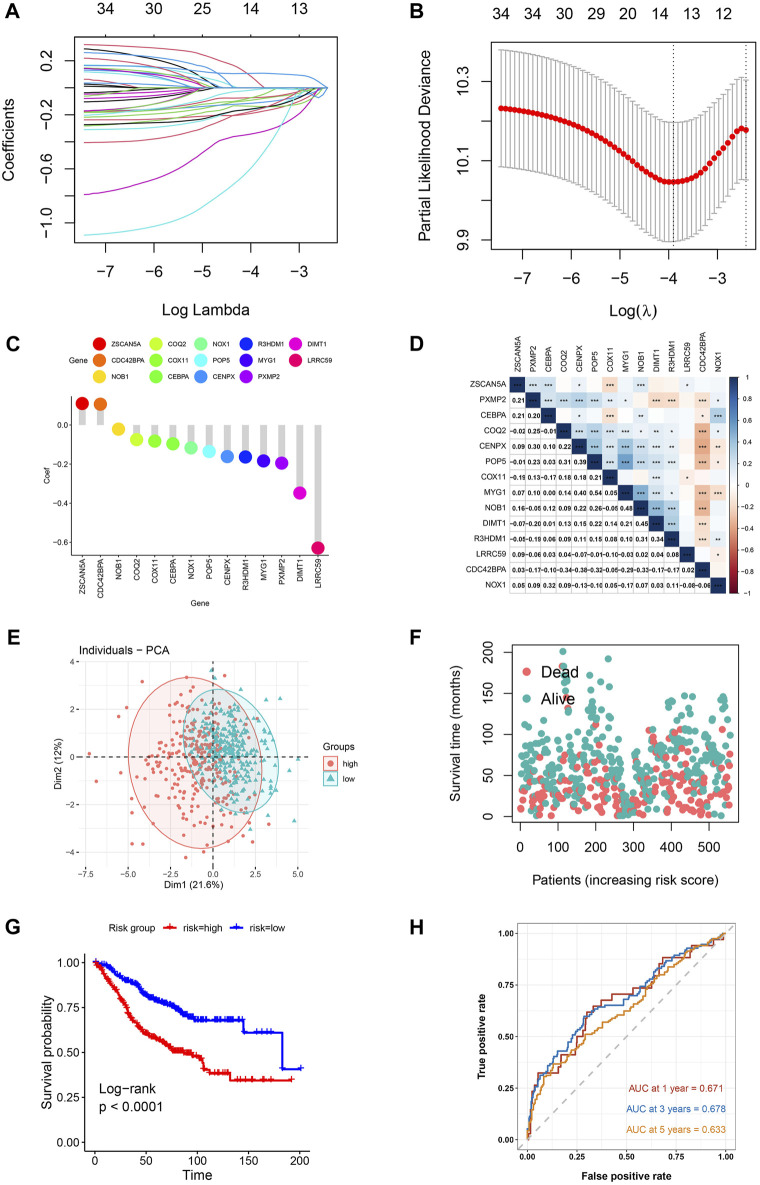
Risk model for CRC patients based on T2DM+CRC mRNAs **(A)** The tuning parameters (log λ) of OS-related proteins were selected to cross-verify the error curve. According to the minimal criterion and 1-se criterion, perpendicular imaginary lines were drawn at the optimal value. **(B)** LASSO coefficient curves and vertical imaginary lines for the 14 OS-related mRNAs were drawn on the values selected for 10-fold cross-validation. **(C)** Coefficient values of T2DM+CRC-related mRNA. **(D)** Spearman correlation analysis of 14 mRNAs (colors represent correlation coefficients, *p*-values of correlation coefficients are marked with an asterisk (*p < 0.05, **p < 0.01, ***p < 0.001)). **(E)** The PCA plot for patients of GSE39582. **(F)** Survival status of each patient. **(G)** Kaplan-Meier curves for the OS. **(H)** Time-dependent ROC curves for OS.

In the training cohort (GSE39582), the prognostic value of every overlapped gene was analyzed utilizing the univariate cox regression model. For further analysis, 34 survival-related genes were identified (*p*-value <0.001): *KIAA1671, PXMP2, CEBPA, NOX1, R3HDM1, DIMT1, NDUFAF2, COQ2, COX11, PAICS, COQ3, CNOT9, POLR2I, ZSCAN5A, POP5, NHP2, RPUSD3, TECR, CENPX, DDX56, SLC37A4, CDC42BPA, LRRC59, IFRD2, NUP85, MTG1, PA2G4, PUS1, NOB1, SNRPF, UTP4, NOP2, POLD2*, and *AURKA*. After constructing a risk signature using the LASSO Cox regression model, 14 genes were screened as per the optimum λ value (Figures 2A, B). Subsequently, the calculation of the risk score as per the coefficient of each gene was executed as mentioned below:

Risk score = −0.195×PXMP2 (exp) + −0.096×CEBPA (exp) + −0.117×NOX1 (exp) + −0.164×R3HDM1 (exp) + −0.348×DIMT1 (exp) + −0.075×COQ2 (exp) + −0.082×COX11 (exp) + 0.110×ZSCAN5A (exp) + −0.135×POP5 (exp) + −0.161×CENPX (exp) + 0.106×CDC42BPA (exp) + −0.629×LRRC59 (exp) + −0.184×MYG1 (exp) + −0.021×NOB1 (exp). (Figure 2C).

Following the exclusion of 23 individuals (lack of corresponding survival data), the remaining participants were then classified as per the median risk score into high-risk and low-risk groups (281 each). Based on the aforementioned risk formula, the risk score of every individual was computed. The association between the 14 mRNAs expressions was assessed by using Pearson correlation coefficients in the GSE39582 cohort (*p < 0.05, **p < 0.01, ***p < 0.001) (Figure 2D). As per the median risk scores obtained, patients in the GSE39582 cohort were classified into groups of low- and high-risk. The principal component analysis indicated the distribution of the two risk groups in different directions (Figure 2E). Based on the distribution graph (Figure 2F), high-risk individuals depicted shorter survival times, and their survival rates also decreased. The Kaplan-Meier curve also depicted a remarkable difference in overall survival across high-risk and low-risk groups, with the high-risk individuals having a remarkably shorter OS time and their survival probability also decreased (HR: 2.338, 95% CI: 1.75–3.11, *p* < 0.001; Figure 2G). The prognosis-predictive accuracy of the risk score was determined by the time-dependent receiver operating characteristic (ROC) curve analysis. The area under the curve (AUC) values for 1-, 3-, and 5-years OS were 0.671, 0.678, and 0.633, respectively (Figure 2H).

### Assessment of clinical characteristics between the high- and low-risk groups

The variations in clinical characteristics between the two groups were examined. Associated analysis depicted that the group with high risk had higher pathological, T- and N-stages, and were older in age in contrast with the group with low risk (Table 2).

**TABLE 2:** Clinical features of two groups in GSE39582.

	High-risk group	Low-risk group	*p* Value
Number	278	278	
Age (median [IQR])	69.00 [60.00,78.00]	68.00 [57.25,74.00]	<0.05
Gender (%)			0.11
Female	115	134	
Male	163	145	
T stage			<0.01
T1	4	7	
T2	13	31	
T3	181	179	
T4	69	48	
Tis	0	4	
NA	11	9	
N stage			<0.01
N0	127	168	
N1	68	63	
N2	63	35	
N3	5	1	
NA	15	11	
M stage			0.53
M0	231	243	
M1	34	26	
NA	13	9	
Pathological stage (%)			<0.001
Stage 0	0	4	
Stage 1	8	24	
Stage 2	119	139	
Stage 3	118	85	
Stage 4	33	26	

### Validation of gene signature in two GEO cohorts and a TCGA cohort

**FIGURE 3:**
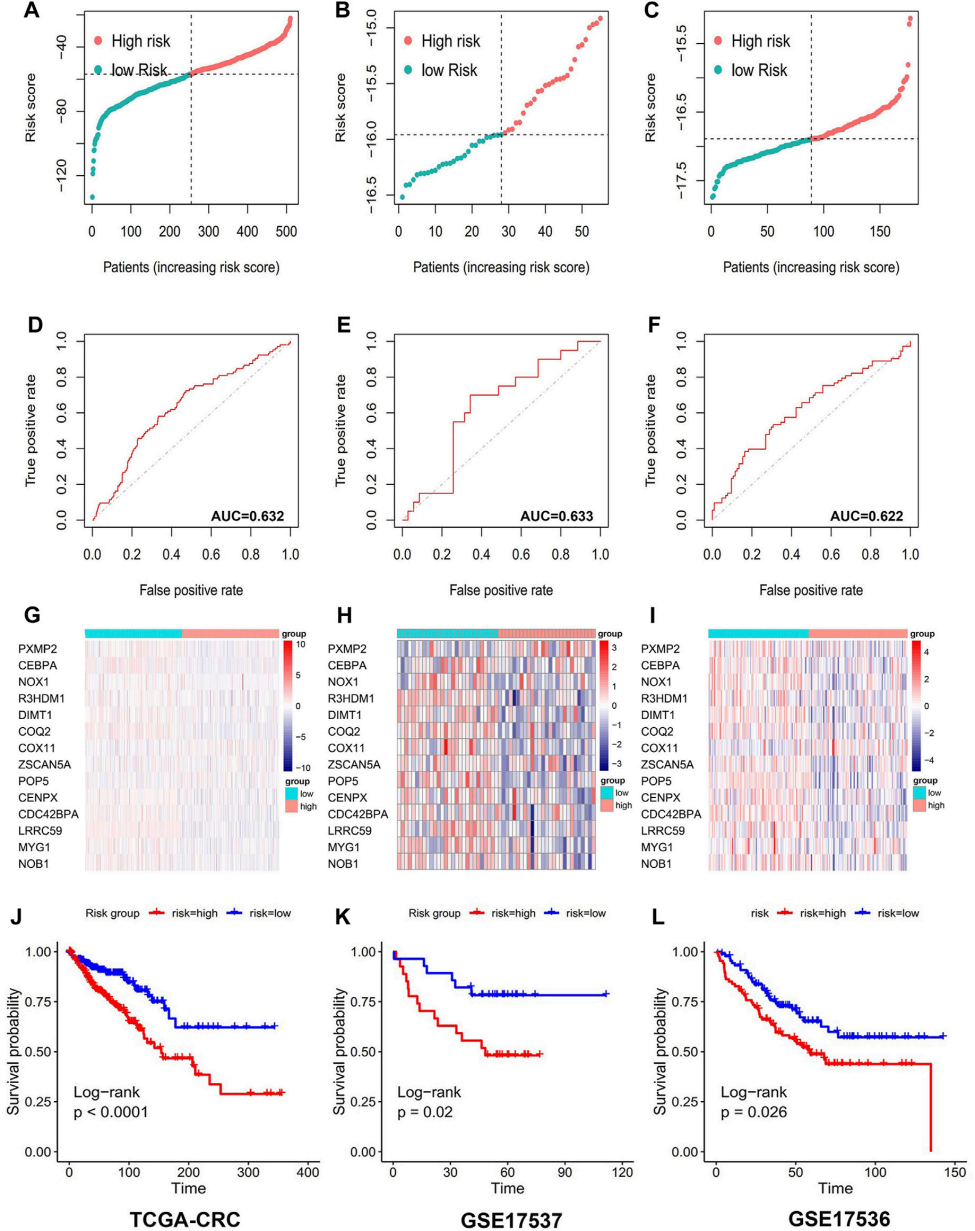
14 Prognostic value of genetic risk models TCGA database and GEO database for T2DM+CRC-associated mRNAs. Distribution of T2DM+CRC-related mRNA-based model risk scores for the validation set cohorts **(A)** TCGA-CRC **(B)** GSE17536 **(C)** GSE 17537; ROC curves show the accuracy of the prognostic model in the validation set cohorts. **(D)** TCGA-CRC **(E)** GSE17536 **(F)** GSE 17537; cluster analysis heat map showing the expression levels of 14 prognostic mRNAs for each patient in the validation set cohorts **(G)** TCGA-CRC **(H)** GSE17536 **(I)** GSE 17537. Kaplan-Meier survival curves for OS of patients in the low- and high-risk groups in the validation set cohorts **(J)** TCGA-CRC **(K)** GSE17536 **(L)** GSE 17537.

T2DM-associated genetic markers were subsequently validated in three external validation sets to determine their stability and generalizability in various populations. Two GEO datasets and the TCGA-CRC dataset were selected as validation sets. Individuals in the TCGA-CRC, GSE17536, and GSE17537 cohorts were also classified into high- or low-risk groups, using the formula used for the GSE39582 cohort. Similar to that in the training group, the risk score distribution (Figures 3A–C) and ROC curves (Figures 3D–F) were analyzed in the three validation cohorts. The results were highly consistent with the GSE39582 cohort. The gene expression heatmap was drawn (Figures 3G–I). The results of the three external validation sets revealed good agreement with the training set data. In the three validation cohorts, Kaplan–Meier analysis predicted better OS in the low-risk groups than in the high-risk groups: GSE17536 (HR: 1.69, 95% CI: 1.07–2.68; *p* < 0.05; Figure 3J), GSE17537 (HR: 2.93, 95% CI: 1.22–7.10, *p* < 0.05; Figure 3K), and TCGA-CRC (HR: 2.30, 95% CI: 1.57–3.37, *p* < 0.001; Figure 3L).

### Comparison of immune infiltration between the two risk groups

**FIGURE 4:**
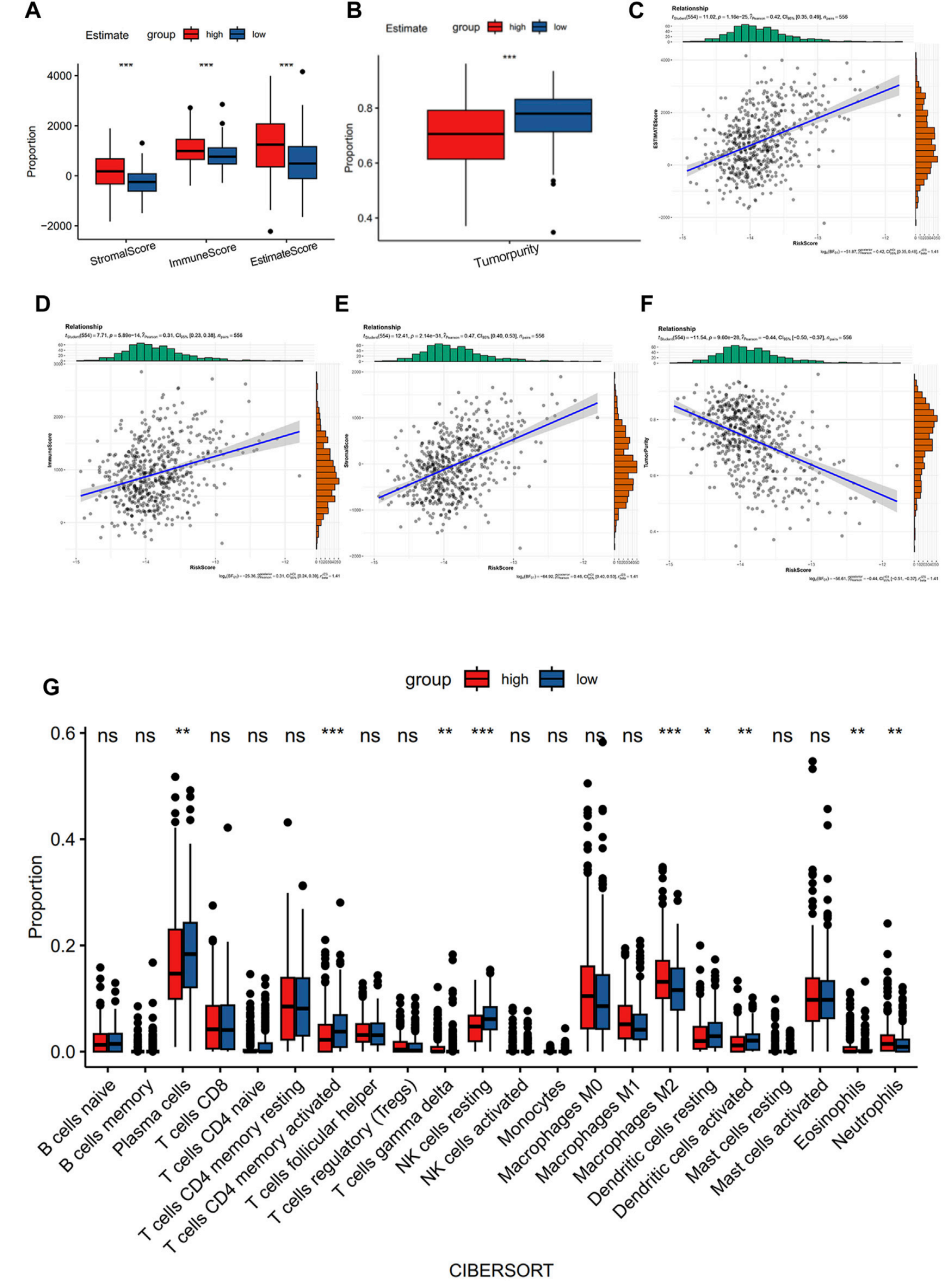
Comparison of immune characteristics between the two groups. **(A)** Correlation analysis of stromal score, immune score, ESTIMATE score, **(B)** tumor purity, ESTIMATE score of CRC patients **(C)**, immune score **(D)**, stromal score **(E)**, and tumor purity **(F)** with risk score. Immune cell ratio **(G)**.

**FIGURE 5:**
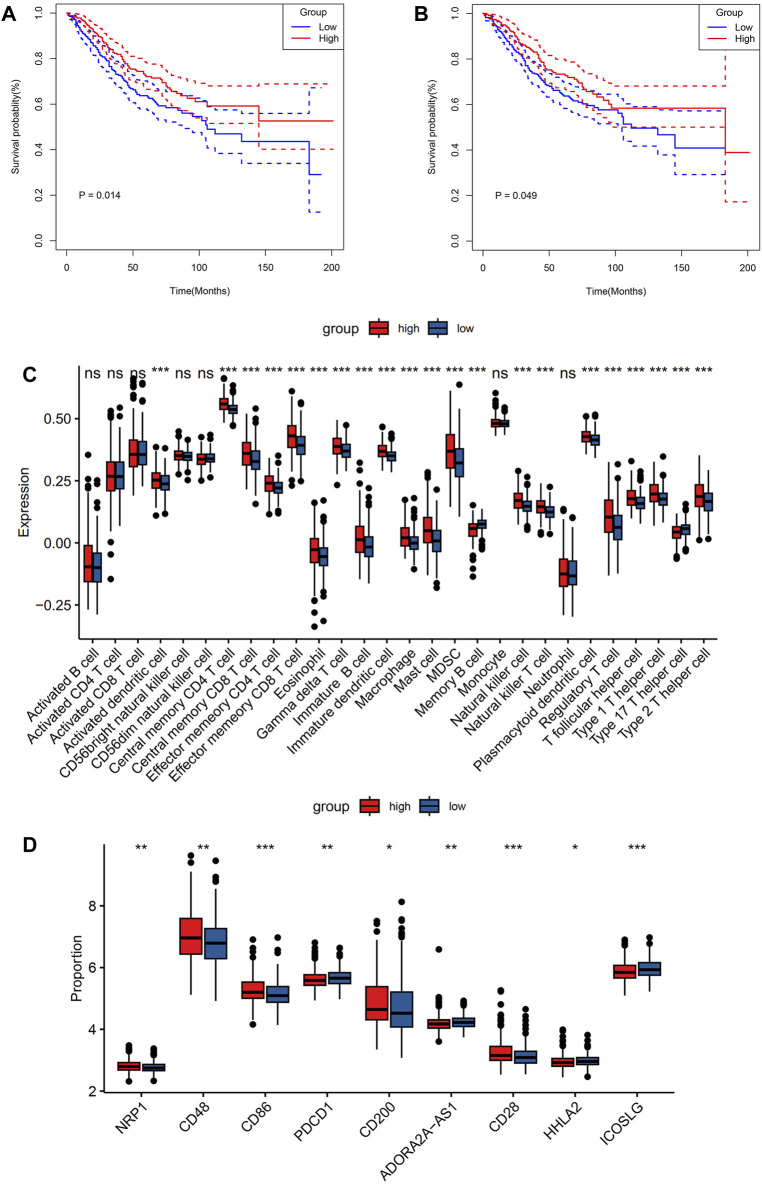
Comparison of immune characteristics between the two groups. Comparison of overall survival for CRC patients with different infiltration levels of NK cells resting **(A)** and T cells CD4 memory activated **(B)** in the GSE39582 cohort. Immune cell expression **(C)**, and immunomodulatory drugs were compared in colorectal cancer clinical trials between the two groups **(D)**. *p* values were marked using asterisks (ns, not significant, *p < 0.05, **p < 0.01, ***p < 0.001).

Using the ESTIMATE algorithm, the variation in the immunological characteristics was explored among individuals with high and low risks of CRC. These finding depicted remarkably elevated stromal, immune, and ESTIMATE scores in the group with high risk than in the group with low risk (all *p* < 0.001) (Figure 4A). In contrast, individuals with a lower CRC risk depicted remarkably increased tumor purity (*p* < 0.001) (Figure 4B). Similarly, the observed association between ESTIMATE, immune, and stromal scores and risk score was positive (all *p* < 0.001) (Figures 4C–E), whereas a negative correlation existed between tumor purity and the risk score (*p* < 0.001) (Figure 4F). CIBERSORT analysis and LM22 single-cell gene expression model matrix were used to compare the infiltration levels of multiple immune cell types between high- and low-risk groups. Additionally, the prognosis-predictive value that these immune cells might hold regarding individuals with CRC was also assessed. The distribution pattern of the expressed 22 immune cell types in CRC patients was examined (Figure 4G). The data indicated that the distribution of the 9 of these types varied considerably between the two groups. Furthermore, these results showed that NK cells resting and T cells CD4 memory activated were significantly associated with a favorable OS in CRC patients (Figures 5A, B). In contrast, higher levels of infiltration of Macrophages M2 and Neutrophils in the high-risk group were strongly linked to adverse clinical outcomes in individuals with CRC (Supplementary Figures S1A, B). The high-risk group showed elevated expression levels of 21 immune cell subtypes, including memory CD4/CD8 T cells (effector and central), activated dendritic cells, natural killer cells, and natural killer T cells, as demonstrated by SSGSEA outcomes. (Figure 5C). Thus, the high-risk group tended to have a higher level of immune infiltration than the low-risk group.

### Association between risk core and immunotherapy response

To assess the sensitivity of individuals with CRC to immunotherapy, the expression levels of several targets of immunomodulatory drugs in metastatic CRC were investigated utilizing clinical trial data. These immunomodulatory targets were compared between the two patient groups in terms of their expression. The resulting data indicated considerably elevated expression levels of most of the immunomodulatory targets (NRP1, CD28, CD48, CD86, and CD200) in the high-risk group (Figure 5D). Hence, this group may exhibit a more positive response to immunotherapy than the low-risk group.

### Results of the nomogram model for predicting survival

**FIGURE 6:**
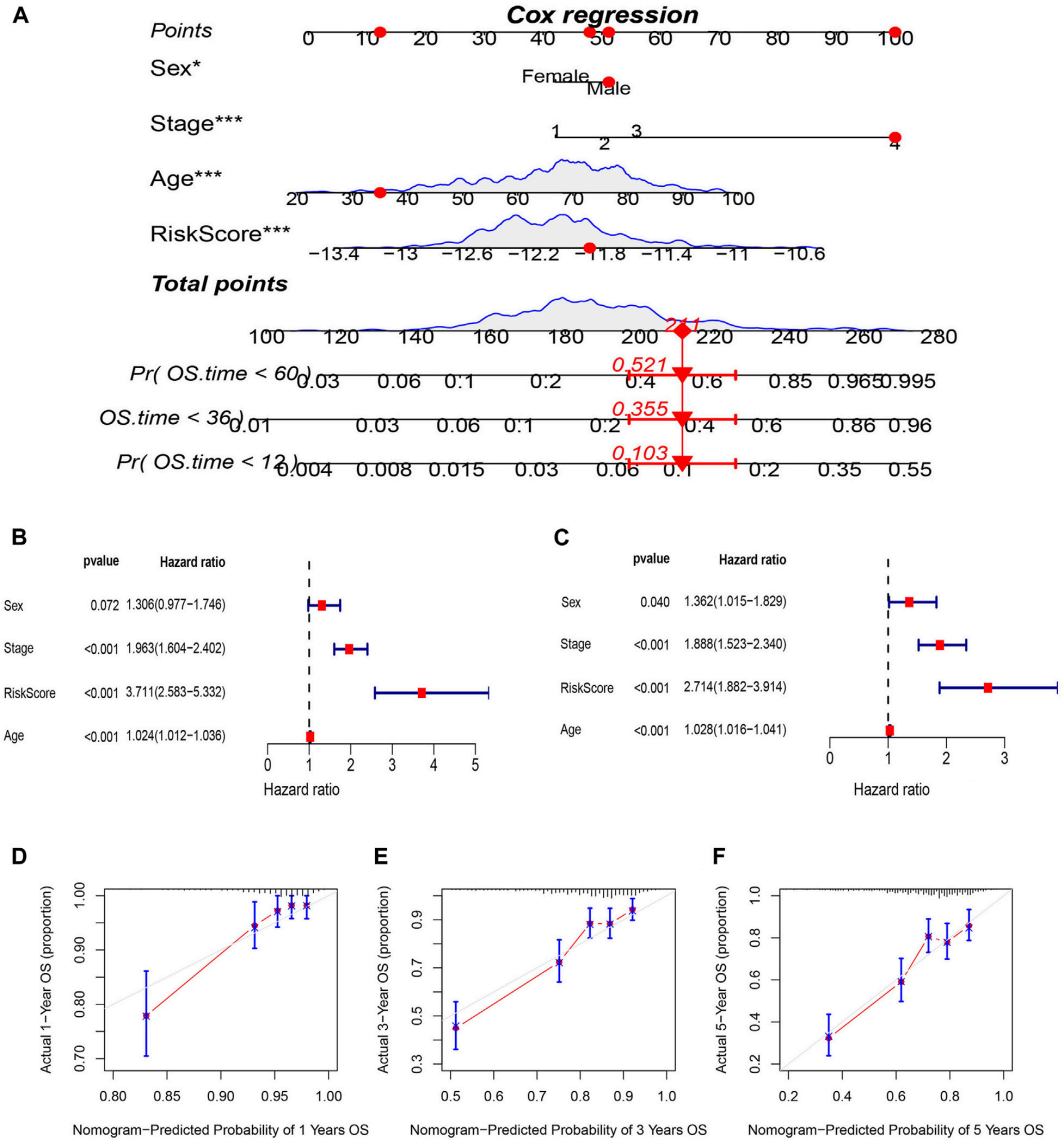
Construction and evaluation of prognostic nomogram. **(A)** nomogram predicting the probability of 1-year, 3-year, and 5-year OS. **(B)** Univariate and **(C)** multivariate analysis of clinical characteristics and risk scores with OS. **(D–F)** Calibration plots of nomogram predicting the probability of 1-year,3-year, and 5-year OS.

A nomogram incorporating age, pathological stage, sex, and the prognostic risk score model was developed to predict OS in CRC patients (Figure 6A). To determine whether the risk score and clinical features could function as independent indicators concerning prognosis, univariate and multivariate Cox regression analyses were performed. The resulting data depicted the capacity of the risk score, age, and TNM stage to independently predict the prognosis in individuals with CRC (Figures 6B, C). A satisfactory concordance was exhibited between the predicted OS rates by the nomogram and observed OS rates by the calibration curves (Figures 6D–F).

### Predicting response to chemotherapy using risk model based on 14 signatures

**FIGURE 7:**
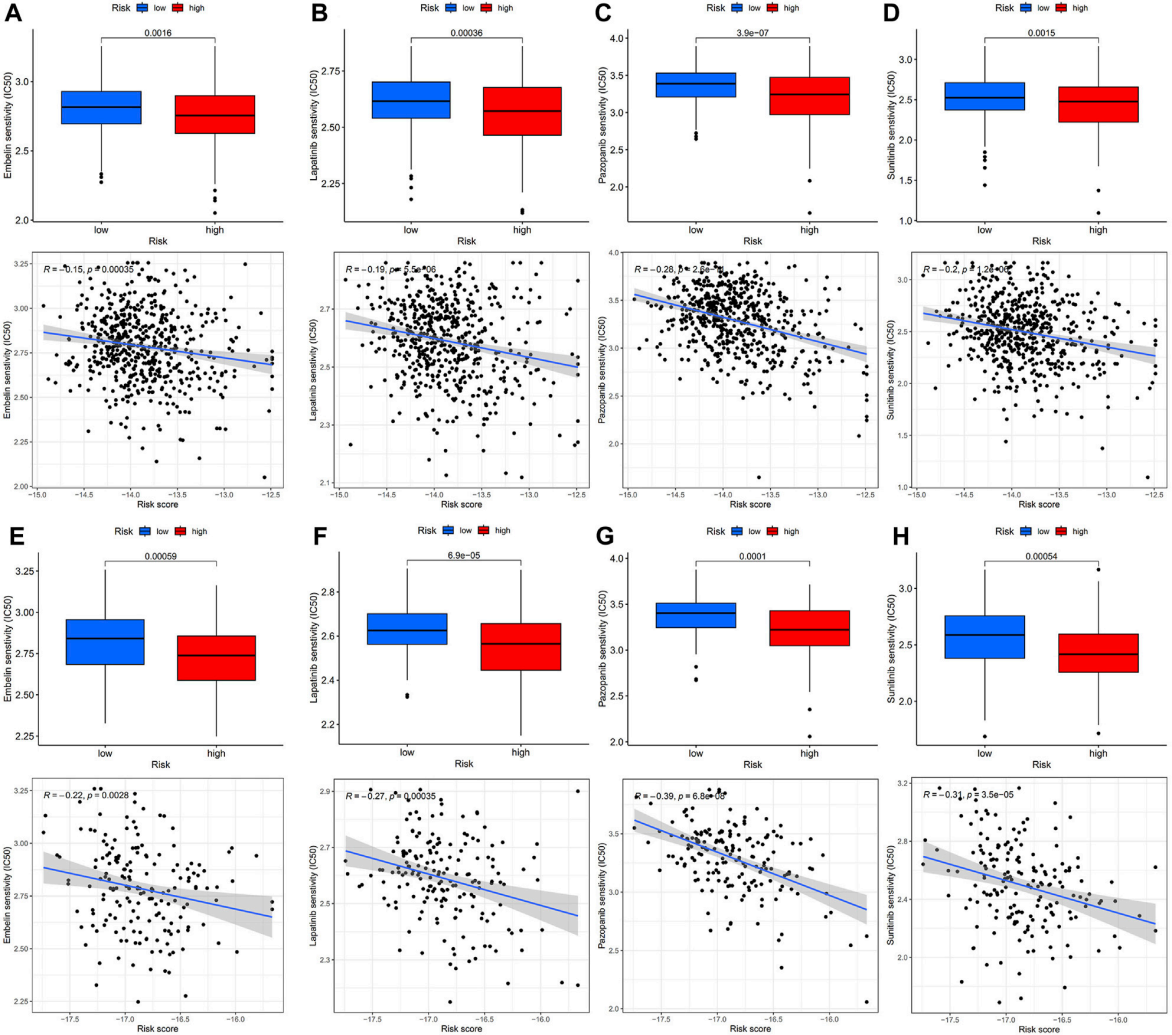
IC50 values of four chemotherapeutic agents with 14-mRNA profiles in two GEO cohorts and the correlation. **(A–D)** Embelin, Lapatinib, Pazopanib and Sunitinib in the GSE39582 cohort. **(E–H)** Embelin, Lapatinib, Pazopanib and Sunitinib in the GSE17536 cohort.

The “pRRophetic” R package was utilized to evaluate the sensitivity of the two risk groups to chemotherapy drugs commonly used for CRC. The resulting data (Figure 7) revealed that the IC50 values of embelin, lapatinib, pazopanib, and sunitinib were considerably reduced in the high-risk group in contrast with the low-risk group, depicting an enhanced sensitivity of the CRC patients in the high-risk group (Figures 7A–D). To further validate these findings, tests were conducted using the GSE17536 dataset, and the results confirmed that the high-risk group had lower IC50 values for embelin, lapatinib, pazopanib, and sunitinib (Figures 7E–H).

### Diagnostic value of hub genes in T2DM-CRC

**FIGURE 8:**
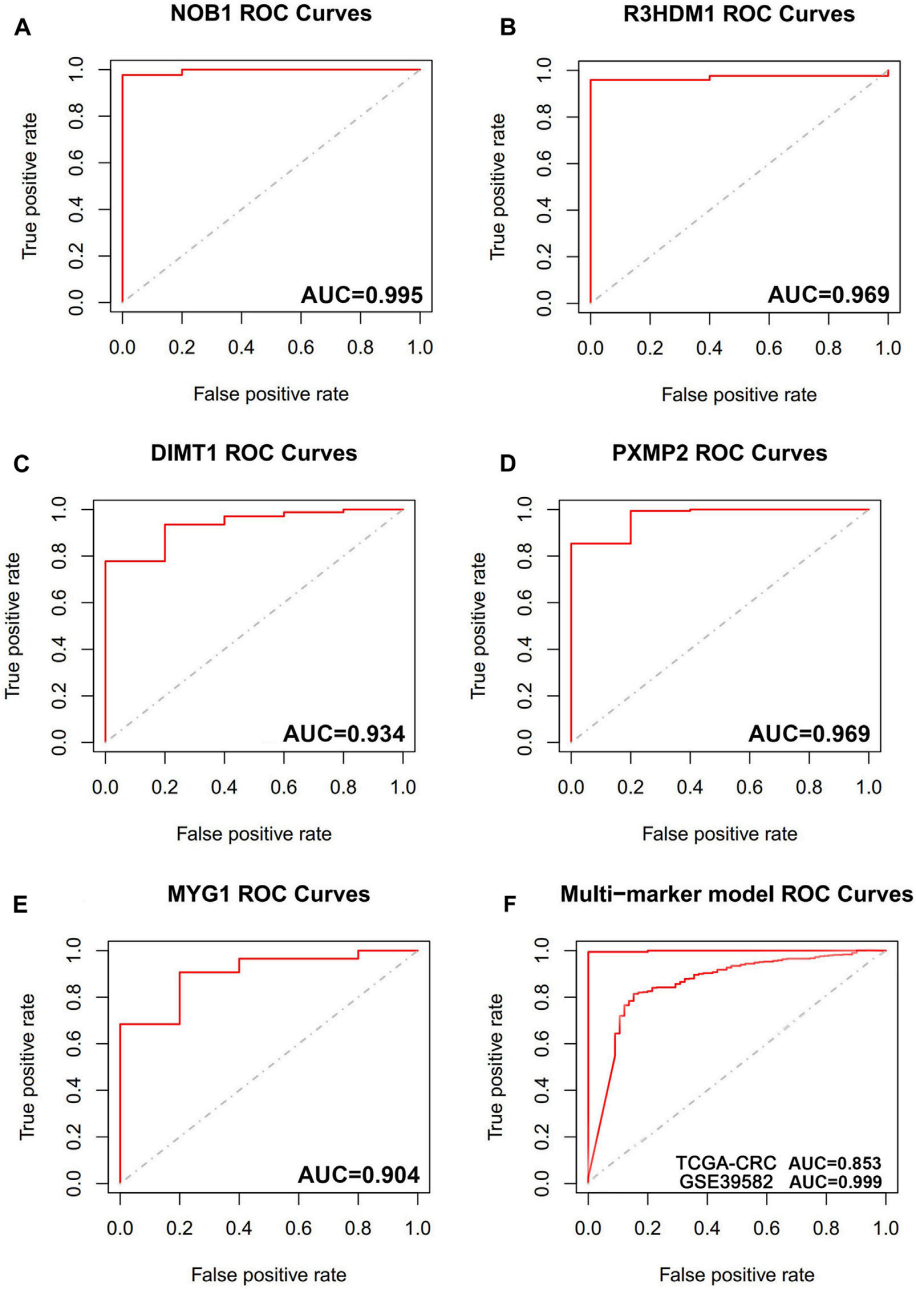
The receiver operating characteristic (ROC) curves of hub genes. **(A)** ROC curve of NOB1. **(B)** ROC curve of R3HDM1. **(C)** ROC curve of DIMT1. **(D)** ROC curve of PXMP2. **(E)** ROC curve of MYG1. **(F)** ROC curve of five genes combination in training cohort (GSE39582), ROC curve of five genes combination in validation cohort (TCGA-CRC).

The diagnostic accuracy of the 14 hub genes was examined through the ROC curve analysis by calculating the AUC values. In total, 5 of 14 hub genes had AUC >0.9, indicating their high diagnostic significance in CRC (Figures 8A–E). To improve their performance regarding their function as predictive indicators, logistics regression analysis was utilized to establish a multi-marker diagnostic model by the integration of these five hub genes. The model was then evaluated using ROC curve analysis, and the data indicated that it had high accuracy and efficiency for diagnosing CRC (AUC = 0.999). The validation of this model in the independent dataset TCGA-CRC showed similar results, with an AUC of 0.853 (AUC = 0.853) (Figure 8F).

## Discussion

The current scientific literature includes many observational studies that have placed patients with T2DM more at risk of CRC (Levi et al., 2002; Coughlin et al., 2004; G et al., 1985). A review of 97 prospective studies linked 123,205 fatalities to CRC in 820,900 patients with T2DM, with a high risk of CRC in individuals with T2DM (RR = 1.40; 95% CI: 1.20–1.63) (Rao Kondapally Seshasai et al., 2011). In addition, other research has depicted an increased incidence of CRC in patients with diabetes in contrast with non-diabetic individuals (Jiang et al., 2011; Deng et al., 2012; Kramer et al., 2012). Moreover, individuals with diabetes have lower OS after CRC (5-year survival rate: 48% vs. 35%) (Barone et al., 2008; van de Poll-Franse et al., 2012). In a previous study where the histopathological features of diabetic and non-diabetic individuals were explored, individuals with diabetes had increased tumor infiltration, higher lymphovascular infiltration, and greater TNM staging (OR [95% CI]: 2.06 [1.37–3.10], 2.52 [1.74–3.63], and 2.45 [1.70–3.52], respectively; *p* < 0.001] (Sharma et al., 2014). These data support the idea that in individuals with diabetes, CRC is more aggressive, requiring appropriate measures to better control diabetes. The relationship between T2DM pathogenesis and CRC progression has been explored in the literature, with several factors being implicated in the increased risk of CRC among individuals with T2DM. These factors include reduced levels of vitamin D, obesity, a sedentary lifestyle, and a high-fat diet (Giovannucci et al., 2010). Abdominal obesity and lack of physical activity are major factors concerning hyperinsulinemia and insulin resistance. Moreover, hyperinsulinemia further increases insulin-like growth factor 1 level, which in turn induces vascular endothelial growth factor expression. This then stimulates tumor cell angiogenesis and promotes tumor cell proliferation, thereby inducing CRC development (Ahmed et al., 2006). Meanwhile, hyperinsulinemia directly stimulates DNA synthesis and growth of normal intestinal epithelial cells and tumor cells in CRC via dose-dependent stimulation, contributing to CRC development and invasion (Ahmed et al., 2006). Furthermore, in animal models, exogenous insulin stimulates the growth of CRC precursors (Jiang et al., 1998). Hence, hyperinsulinemia likely mediates the effect of type 2 diabetes on CRC risk. In addition, chronic hyperglycemia has been reported to significantly increase reactive oxygen species production, increase chronic oxidative stress (Robertson, 2004), and induce inflammatory pathways (Lin et al., 2005). Inflammation has been considered a potential mechanism leading to an enhanced risk of cancerous growth (Rinaldi et al., 2008). Numerous studies have confirmed that patients with diabetes have significant cellular immune dysfunction with a dysregulated ratio of T-lymphocyte subsets (Frolov et al., 1994). Remarkably, decreased immune function can cause tumor cells to escape immune surveillance and survive, inducing malignant tumor development and disease progression. However, the molecular mechanisms underlying the complex interactions between CRC and T2DM are yet to be elucidated.

As far as we are aware, this research is the first to assist in early detection, better therapeutic options, and timely prevention of CRC based on the common genes and features of CRC and T2DM identified using WGCNA. First, individuals with CRC were classified into high- and low-risk groups as per the intermediate-risk scores, with considerably poorer clinical outcomes being assigned to the former group. Multiple Cox regression analysis indicated that the 14-gene model was an independent risk factor for OS. Moreover, ROC analysis indicated increased efficacy of the model in contrast with the conventional clinical features in predicting survival in CRC patients. Additionally, a nomogram that depicted congruence between the nomogram-predicted and observed rates of 1-, 3-, and 5-year OS was developed. Traditionally, the prognosis of individuals with CRC relies on TNM staging, which, although valuable, has limitations due to the lack of data regarding the cellular and molecular levels. This may cause the clinical outcomes of individuals with CRC who have the same TNM stage to vary considerably. Therefore, several studies have focused on assessing a single biomarker as the prognostic indicator for CRC along with various other tumors. Moreover, integrating multiple elements into a prognostic model has been shown to considerably improve the prognostic value of a single biomarker in various malignancies (Halabi et al., 2013; Zhang et al., 2013; Cardoso et al., 2016). Accordingly, the current research developed a new prognostic model to effectively assess the risk score and predict the prognosis of individuals with CRC.

Subsequently, the study comparatively assessed the immunological features of the two aforementioned groups using CIBERSORT, SSGSEA, and ESTIMATE. The ESTIMATE data indicated that the group with increased risk depicted elevated immune, stromal, and ESTIMATE scores in contrast with the group with low risk, suggesting a more active tumor immune microenvironment in the group with high risk. Remarkably, the extent of immune cell infiltration into tumors has been associated with the growth of the tumor, its progression, and prognosis, making these areas crucial for research recently (Jass, 1986; Ogino et al., 2009). CIBERSORT, a biological software, can assess the expression profiles of complex tissues for the purpose of examining the immune cell composition (Newman et al., 2015). Therefore, in this study, CRC-related genes in the risk groups were assessed through CIBERSORT. In the GSE39582 dataset, the number of CD4 resting memory T cells was increased in the group with low risk. Prior research has documented that CD4 memory T cells may inhibit the progression of tumors by upregulating the proliferative ability of CD8T cells, which differentiate into effector cells following their migration into tumor-associated tissues (Crouse et al., 2015). A report on breast cancer has demonstrated that an elevated level of resting and activated CD4 memory T cells is directly linked to greater disease-free survival (Zhang et al., 2019). Macrophages perform a vital function in tumor progression with earlier and more numerous immune cell infiltration in TME. Following induction by different cytokines, macrophages develop into their subtypes M0, M1, and M2, each with various immune functions (Mosser and Edwards, 2008). M0 is an inactivated subtype with no inflammatory or tumor-associated functions. Depending on the pathway employed for activation, M0 can differentiate into two activated subtypes M1 and M2 that exhibit different immunomodulatory effects. M1 macrophages release pro-inflammatory cytokines, such as interleukin (IL)-12, IL-16, interferon-γ, and tumor necrosis factor-α; they thus activate inflammatory responses and contribute to the innate immunity of the host, eventually destroying the cells in TME (Galdiero et al., 2013). M2 macrophages are primarily involved in the secretion of cytokines such as IL-10 and transforming growth factor-β to suppress inflammation (Sica and Mantovani, 2012). Such cells participate in Th2-type immune response, inhibiting the proliferative and differentiation capacity of T cells and promoting tumor cell proliferation and tumor stromal angiogenesis (Chen et al., 2017; Martinez et al., 2017). The CIBERSORT analysis implied that the level of M2 macrophages was increased in the group with high risk than in the group with low risk. This suggested that the high-risk group could tend to establish a tolerogenic TME. Zhao et al. linked the promotion of liver metastasis in CRC to M2 macrophage polarization (Zhao et al., 2020). Based on the findings of SSGSEA, the expression level of 25 immune cell subtypes was considerably upregulated in the high-risk group; these subtypes included macrophages, natural killer T (NKT) cells, CD4 cells, natural killer (NK) cells, and dendritic cells (DC). A strong impact of the tumor-infiltrating T cells was observed on the clinical outcome of individuals with CRC. The increased CD8 T cell infiltration enables the prediction of sensitivity to drugs and improves survival in individuals with CRC and liver metastases (Galon et al., 2006; Fridman et al., 2012). Prior research on CRC has demonstrated a better prognosis in individuals with elevated Th1 levels, while individuals with upregulated Th17 have a poorer prognosis. Furthermore, the influence of Th1 on survival appears to outweigh the impact of Th17 (Tosolini et al., 2011). Reportedly, the major antigen-presenting cells, the DCs, activate T cells, thereby promoting antitumor immunity (Wculek et al., 2020). In addition, following NK cell stimulation, the conventional type 1 DCs are recruited into the TME (Bottcher et al., 2018). NK cells cause immune-induced cytotoxicity in tumor cells; therefore, its increased infiltration results in a better outcome in CRC (Coca et al., 1997). Furthermore, the vital role of NKT cells in anti-tumor immunity was revealed in anti-PD-1 resistant tumor models wherein these cells exhibited the ability to reinvigorate the depleted CD8 T cells (Bae et al., 2018). Prior research depicted that elevated levels of NKT cell infiltrates can independently function as a CRC prognostic factor (Tachibana et al., 2005). Based on our study of immune texture, the high-risk group had a more extensive immune cell infiltration than the low-risk group. Hence, the former group may possess better immunological competence, making them better candidates for immunotherapy.

The CGP contains data regarding drug sensitivity and molecular markers of drug response in cancer cells and is a public resource. Currently, data regarding cancer cell lines (almost 700 in total) can be accessed at the CGP regarding their response to 251 chemotherapeutic drugs. To design an improved treatment plan for CRC, pharmacosensitivity analysis was conducted on high- and low-risk populations. The resulting data indicated four drugs with potential clinical significance, including embelin, lapatinib, pazopanib, and sunitinib. In the future, the risk score of CRC patients can be identified based on this risk model and a more appropriate therapeutic schedule can be developed. This will help further improve chemotherapy efficacy and reduce drug resistance.

In summary, a T2DM-related CRC risk score model was developed and validated in this research. This model can accurately predict OS and sensitivity to common chemotherapeutic agents in CRC patients. In addition, a 5-gene signature diagnostic model was identified, and an external validation set was utilized to validate the model. Furthermore, the characteristics of immune cell infiltration and TME were assessed. This may help in understanding immune mechanisms and may contribute to the treatment of T2DM-related CRC as well as the assessment of prognosis.

## Data Availability

The original contributions presented in the study are included in the article/Supplementary Material, further inquiries can be directed to the corresponding author.
