# Supplementary material for: Identification of the shared gene signature and biological mechanism between type 2 diabetes and colorectal cancer
Source: Front Genet. 2023 Oct 9;14:1202849. doi: 10.3389/fgene.2023.1202849 (PMC10593476; doi:10.3389/fgene.2023.1202849)
Supplement: Supplementary file 1 [file Table1.DOCX]

Supplementary Material

Identification of the shared gene signature and biological mechanism between type 2 diabetes and colorectal cancer

Xianqiang Liu^1,2, †^, [Dingchang Li](https://www.frontiersin.org/people/u/1465938)^1,2, †^, Wenxing Gao^1,2, †^, Wen Zhao^2,3^, Lujia Jin^1,2,^ Peng Chen^1,2^, Hao Liu^1,2^, Yingjie Zhao^1,2^ and Guanglong Dong^2*^

1 Medical School of Chinese PLA, Beijing, 100853, China

2 Department of General Surgery, The First Medical Center, Chinese PLA General Hospital, Beijing, 100853, China.

3 School of Medicine, Nankai University, Tianjin, 300071, China;

Electronic address: dongguanglong@301hospital.com.cn.

# Supplementary Figures and Tables

## Supplementary Figures


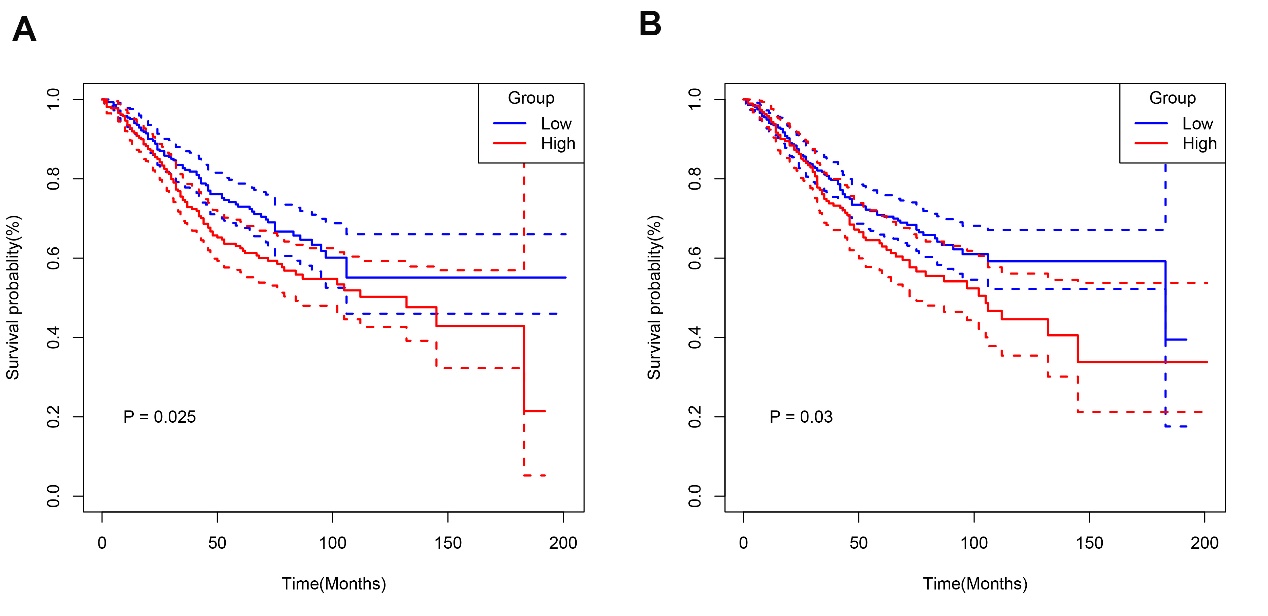


**Supplementary Figure 1.** Kaplan-Meier survival curves of overall survival in CRC patients. Comparison of overall survival for CRC patients with different infiltration levels of Macrophages M2 (A) and Neutrophils(B) in the GSE39582 cohort, respectively. CRC, colorectal cancer
